# The difference between ‘placebo group’ and ‘placebo control’: a case study in psychedelic microdosing

**DOI:** 10.1038/s41598-023-34938-7

**Published:** 2023-07-26

**Authors:** Balázs Szigeti, David Nutt, Robin Carhart-Harris, David Erritzoe

**Affiliations:** 1 Centre for Psychedelic Research, Imperial College London, London, UK; 2 Psychedelics Division, Neuroscape, Department of Neurology, University of California San Francisco, San Francisco, USA

**Keywords:** Computational biology and bioinformatics, Neuroscience, Psychology, Medical research

## Abstract

In medical trials, ‘blinding’ ensures the equal distribution of expectancy effects between treatment arms in theory; however, blinding often fails in practice. We use computational modelling to show how weak blinding, combined with positive treatment expectancy, can lead to an uneven distribution of expectancy effects. We call this ‘activated expectancy bias’ (AEB) and show that AEB can inflate estimates of treatment effects and create false positive findings. To counteract AEB, we introduce the *Correct Guess Rate Curve (CGRC)*, a statistical tool that can estimate the outcome of a perfectly blinded trial based on data from an imperfectly blinded trial. To demonstrate the impact of AEB and the utility of the CGRC on empirical data, we re-analyzed the ‘self-blinding psychedelic microdose trial’ dataset. Results suggest that observed placebo-microdose differences are susceptible to AEB and are at risk of being false positive findings, hence, we argue that microdosing can be understood as active placebo. These results highlight the important difference between ‘*trials with a placebo-control group*’, i.e., when a placebo control group is formally present, and ‘*placebo-controlled trials*’, where patients are genuinely blind. We also present a new blinding integrity assessment tool that is compatible with CGRC and recommend its adoption.

## Introduction

In medical research the gold standard experimental design is the blinded randomized controlled trial^1^, where ‘blinding’ refers to the concealment of the intervention^2^. The purpose of blinding is to equally distribute expectancy effects between treatment arms^3^, thus, to eliminate biases associated with expectancy. ‘Blinding integrity’ refers to how successfully blinding is maintained. Blinding integrity can be assessed by asking blinded parties, e.g., patients and/or doctors, to guess treatment allocation. If the correct guess rate (CGR) is higher than chance, then, blinding is ineffective. Assessing blinding integrity could be especially important when outcomes are subjective, for example in pain and psychiatric research, where there is a high susceptibility to expectation biases^4^. In these domains, only 2–7% of trials report blinding integrity and when blinding is assessed, it is found to be ineffective for about 50% of the trials^5–9^.

Poor reporting of blinding integrity may be explained by at least three factors. First, there is no accepted standard for how to assess blinding integrity. Most commonly, patients are asked to guess their treatment after the trial has concluded, but such data may be subject to recall and other biases^10–12^. Secondly, there is no accepted standard for how to incorporate blinding integrity into data analysis. Even if blinding integrity is assessed, most scientific reports do not attempt to incorporate blinding integrity data into the interpretation of the results. Finally, others have speculated that a reluctance to assess blinding stems from a fear that weak blinding could cast doubt on positive trial outcomes^5^. Supporting this reasoning, lesser blinding integrity reporting has been associated with industry sponsorship^6,9^.

There is a resurgent interest in the medicinal potential of psychedelic drugs, such as LSD and psilocybin^13^. Recently, ‘microdosing’ has emerged as a new paradigm for psychedelic use. Microdosing does not have a universally accepted definition, but most microdosers take oral doses of 10–20 μg LSD or 0.1–0.3 g of dried psilocybin containing mushrooms, 1–4 times a week^14^. Anecdotal claims have been made that microdosing improves well-being and cognition^15,16^. Observational studies have generally confirmed the positive anecdotal claims^17–20^, but so far placebo-controlled studies have failed to find robust evidence for larger than placebo efficacy in healthy samples^21–25^.

We recently conducted a ‘self-blinding citizen science trial’ on microdosing, where participants implemented their own placebo control based on online setup instructions without clinical supervision^24^. The strength of this design is twofold: it tested the effects of microdosing in a real-life context, increasing the trial’s external validity^26^, and it allowed us to obtain a large sample size while implementing placebo control at minimal logistic and economic costs. The study was completed by 191 participants, making it the largest placebo-controlled trial on psychedelic microdosing for a fraction of the cost of even a small traditional clinical trial.

## Methods

### Activated expectancy bias (AEB) model

**Figure 1:**
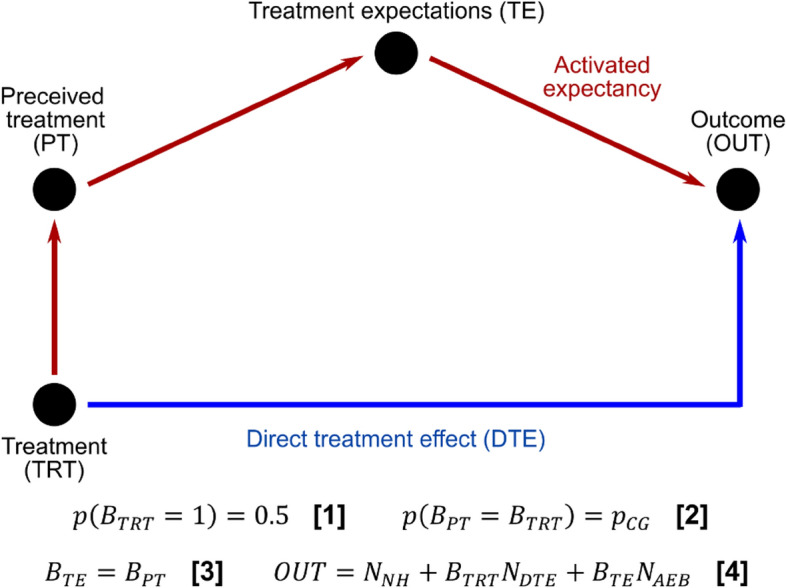
The activated expectancy bias (AEB) model, consisting of 3 binary nodes (TRT, PT and TE) and a continuous value node, the outcome (OUT). In the equations, ${B}_{X} / {N}_{X}$ stand for a random Bernoulli/normal variable, respectively. The binary nodes (TRT, PT and TE) represent Bernoulli variables (B_TRT_, B_PT_, B_TE_), where the values of 0/1 correspond to placebo/active. To generate AEB model data, first *Treatment* (TRT) is determined by Eq. 1 and then the *Perceived treatment* (PT) by Eq. 2, where ${p}_{CG}$ is the probability of correct guess, i.e. the correct guess rate, and then *Treatment expectancy* is fixed according to Eq. 3. Finally, the outcome score is calculated by Eq. 4 which has components of natural history (${N}_{NH}$), direct treatment effect (${N}_{DTE}$) and activated expectancy bias (${N}_{AEB}$), see Supplementary table 1 for the numeric value of all parameters.

We introduce a theoretically motivated computational model of AEB, the model’s structure and equations are shown on Fig. 1, the key model features are:

- The presence or lack of side effects allow patients to infer their treatment at a higher than chance rate. The correct guess probability, ${p}_{CG}$, in the model is 0.7, which is consistent with both microdosing^21,24^ and antidepressants^27–29^ trials.
- AEB model parameters are calibrated such that the treatment effect is 3 points, corresponding to a small-moderate effect size of 0.4 standardized mean difference, which is consistent with microdosing^21,22,24^ and antidepressant trials^30^, numeric parameters can be found in Supplementary Table 1.
- Patients have higher efficacy expectations for the active treatment than for placebo treatment, this positive expectancy bias is represented by the ${N}_{AEB}$ term in the model, see Fig. 1.

The AEB model was used to generate pseudo-experimental data with 2*2 = 4 parameter configurations, corresponding to *direct treatment effect* and *activated expectancy bias* being either active or not, see Fig. 1. In our analysis the direct treatment effect (blue)/activated expectancy bias (red) pathways are turned off by setting the mean ${N}_{DTE}$/${N}_{AEB}$ equal to 0. For each configuration, 500 trials were simulated, each with 230 patients, mimicking the sample size of the microdose trial analyzed.

### Self-blinding microdose trial

The self-blinding microdose trial used an 'self-blinding' citizen science approach, where participants implemented their own placebo control based on online setup instructions without clinical supervision^24^. Self-blinding involved enclosing the microdoses inside non-transparent gel capsules and using empty capsules as placebos. Then, these capsules were labeled with QR codes that allowed investigators to track when placebo/microdose was taken without sharing this information with participants. Participants were followed throughout a 4-week dosing period, taking 2 microdoses/week in the active group. For each capsule taken, participants made a binary guess whether their capsule was placebo or microdose, see Supplementary materials for details.

Here, the trial’s acute and post-acute outcomes are re-analyzed. Acute measures were completed 2–6 h after ingestion of the capsule, while post-acute measures were taken the day after a capsule was taken. Acute outcomes were: *positive and negative affect schedule* (PANAS)^31^, *cognitive performance score* (CPS) and visual analogue scale items for *mood*, *energy*, *creativity*, *focus*, and *temper*. The CPS is an aggregated quantification of cognitive performance based on 6 computerized tasks (*spatial span, odd one out, mental rotations, spatial planning, feature match, paired associates*). Post-acute outcomes were: Warwick–Edinburgh mental well-being scale (WEMWB)^32^, quick inventory of depressive symptomatology (QIDS)^33^, state-trait anxiety inventory (STAIT)^34^ and social connectedness scale (SCS)^35^. To simplify the current analysis, we only used data from the first week of the experiment, thus, each datapoint is independent and not confounded by order effects. This approach reduced the overall sample, but yielded almost identical qualitative conclusion as the full dataset. In the current analysis n = 233 datapoints were included.

The trial only engaged people who planned to microdose through their own initiative, but who consented to incorporate placebo control to their self-experimentation. The trial team did not endorse microdosing or psychedelic use and no financial compensation was offered to participants. The study was approved by Imperial College Research Ethics Committee and the Joint Research Compliance Office at Imperial College London (reference number 18IC4518). Informed consent was obtained from all subjects, the trial was carried out in accordance with relevant guidelines and regulations.

### Estimate of treatment effects

Throughout this work treatment effects are estimated by an *outcome* ~ *treatment* linear model, where *outcome* is a numeric, *treatment* is a binary variable (placebo or active treatment). In this manuscript ‘non-CGR adjusted analysis’ means that this model is fitted to empirical data, while ‘CGR adjusted analysis’ means that this model is fitted to the CGR adjusted pseudo-experimental data, see *Correct guess rate curve* section for details. Therefore, the CGR-adjusted treatment estimate/p-value is to the estimate/p-value associated with the *treatment* term in the model above, applied to data adjusted by the CGRC method. All linear models were implemented using the lme package (version 3.1–155) in R (v4.0.2).

### Correct guess rate curve

**Figure 2:**
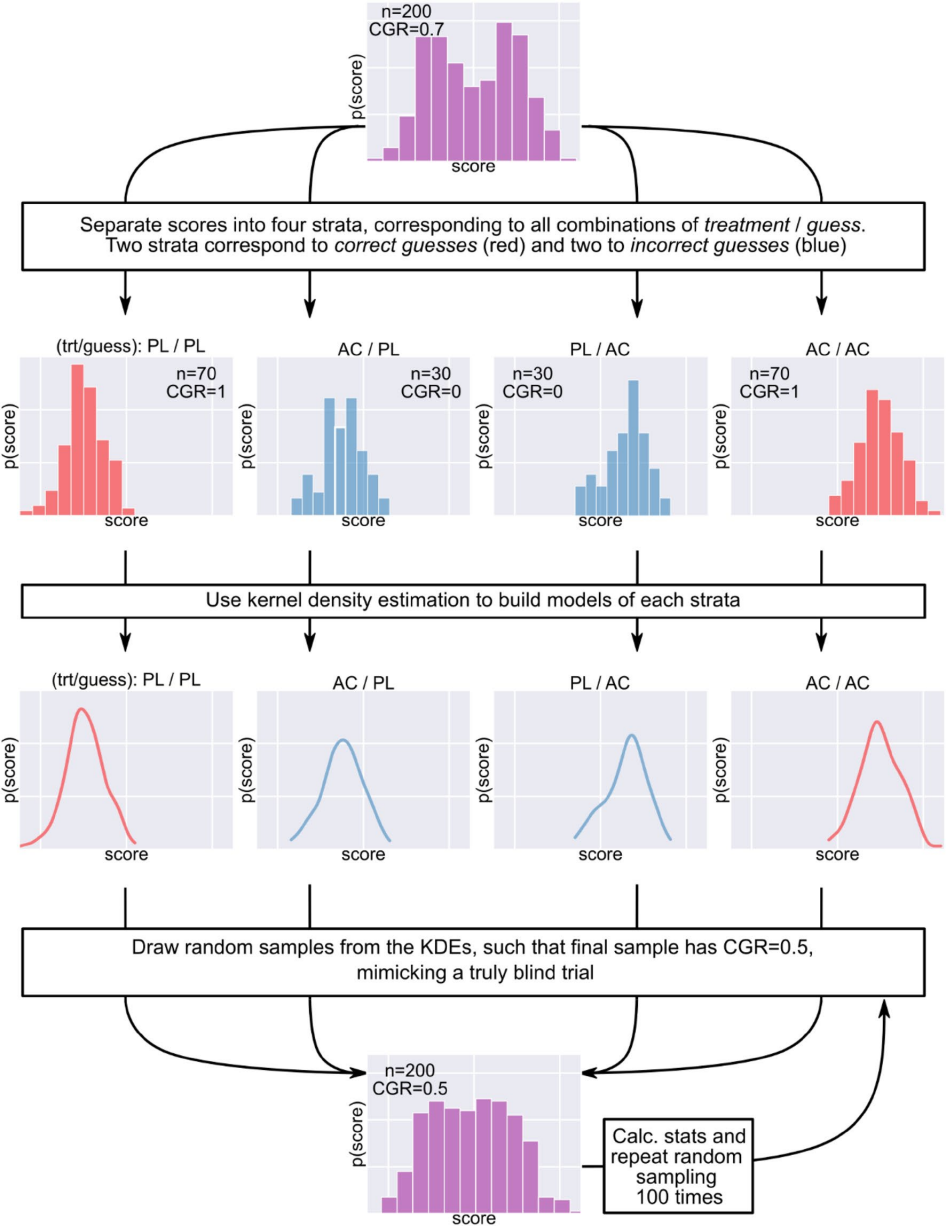
Correct guess rate (CGR) adjustment to estimate the outcome of a perfectly blinded trial based on data from an imperfectly blinded trial. First, scores (purple histogram at top) are separated into four strata corresponding to all possible combinations of *treatment* and *guess*. Both *treatment* and *guess* are binary with potential values of placebo/active, thus, the four strata are (using the *treatment*/*guess* notation): PL/PL, AC/PL, PL/AC and AC/AC. Next, statistical models of these strata are built using kernel density estimation (KDE). Note that two strata correspond to correct guesses (PL/PL and AC/AC; red) and two to incorrect guesses (AC/PL, PL/AC; blue). Next, n/2 random samples are drawn from the correct guess KDEs, such that the relative sample sizes of the correct guess strata are preserved, i.e. the ratio n_PL/PL_/n_AC/AC_ is same as in the original data, see Supplementary materials for a numeric example. Similarly, n/2 random samples are drawn from the incorrect guess KDEs, such that the ratio n_AC/PL_/n_PL/AC_ is same as in the original data. These random samples are then combined, resulting in a pseudo-experimental dataset with CGR = 0.5 (purple distribution at bottom), corresponding to effective blinding. The random sampling from KDEs is repeated 100 times, for each CGR-adjusted pseudo-experimental dataset is analyzed to estimate the direct treatment effect, see *Estimate of treatment effects*. The ‘CGR adjusted treatment effect/p-value’ is the mean treatment estimate / p-value across these 100 samples. Estimates at other CGR values can be obtained similarly, e.g. a trial with CGR = 0.6 can be approximated by drawing 0.6*n random samples from the correct guess KDEs and 0.4*n random samples from the incorrect guess KDEs, etc.

We developed *CGR adjustment,* a novel statistical technique that can estimate the outcome of a perfectly blinded trial, based on data from an imperfectly blinded trial. Briefly, first the scores are separated into four strata corresponding to all four possible combinations of *treatment* and *guess*. Next, statistical models of these four strata are built using kernel density estimation (KDE). KDE estimates were implemented by the scikit-learn package (v1.0.2) in python (v3.7), all parameters were left at default value. Then, random samples are drawn from each strata, such that the combined sample has CGR = 0.5, mimicking a perfectly blinded trial, see Fig. 2 for a detailed explanation. Treatment estimates for other CGR values can be obtained in a similar manner by changing the number of samples drawn from each KDE. For example, a trial with CGR = 0.6 can be approximated by drawing 0.6*n random samples from the *correct guess* KDEs and 0.4*n random samples from the *incorrect guess* KDEs, etc.

## Results

### Correct guess rate (CGR) adjustment of the activated expectancy bias (AEB) model

We analyze pseudo-experimental data generated by the 2*2 = 4 configurations of the AEB model (corresponding to *direct treatment effect* and *activated expectancy bias* either being active or not, see Fig. 1) with both traditional, i.e. non-CGR adjusted, and CGR-adjusted analysis. To demonstrate that the qualitative conclusions presented here do not require fine tuning of parameters, we present a robustness analysis in the Supplementary Materials.

First, the case was analyzed where neither *direct treatment effect* nor the *activated expectancy bias* pathways are activated (top row in Table 1). In this case, the outcome is a normal random variable. The *treatment* p-value was significant for 5%/6% of the simulated trials using the traditional/CGR adjusted models, which is expected based on the 0.05 significance level.

**Table 1:** Comparative results of traditional and CGR adjusted analysis of the AEB model.

Model configuration	Direct treatment effect (points)	Direct treatment effect (Hedges’ g)	Non-CGR adjusted models			CGR adjusted models		
			Average treatment p-value	Proportion with sig. treatment p-value	Average treatment effect (points)	Average treatment p-value	Proportion with sig. treatment p-value	Average treatment effect (points)
DTE off, AEB off	0	0	0.503	0.05	0.0	0.332	0.06	0.02
DTE on, AEB off	3	0.4	0.032	0.86	3.02	0.036	0.84	3.01
DTE off, AEB on	0	0	0.052	0.78	2.91	0.381	0.03	0.01
DTE on, AEB on	3	0.4	0.001	0.99	5.69	0.041	0.82	3.04

**Figure 3:**
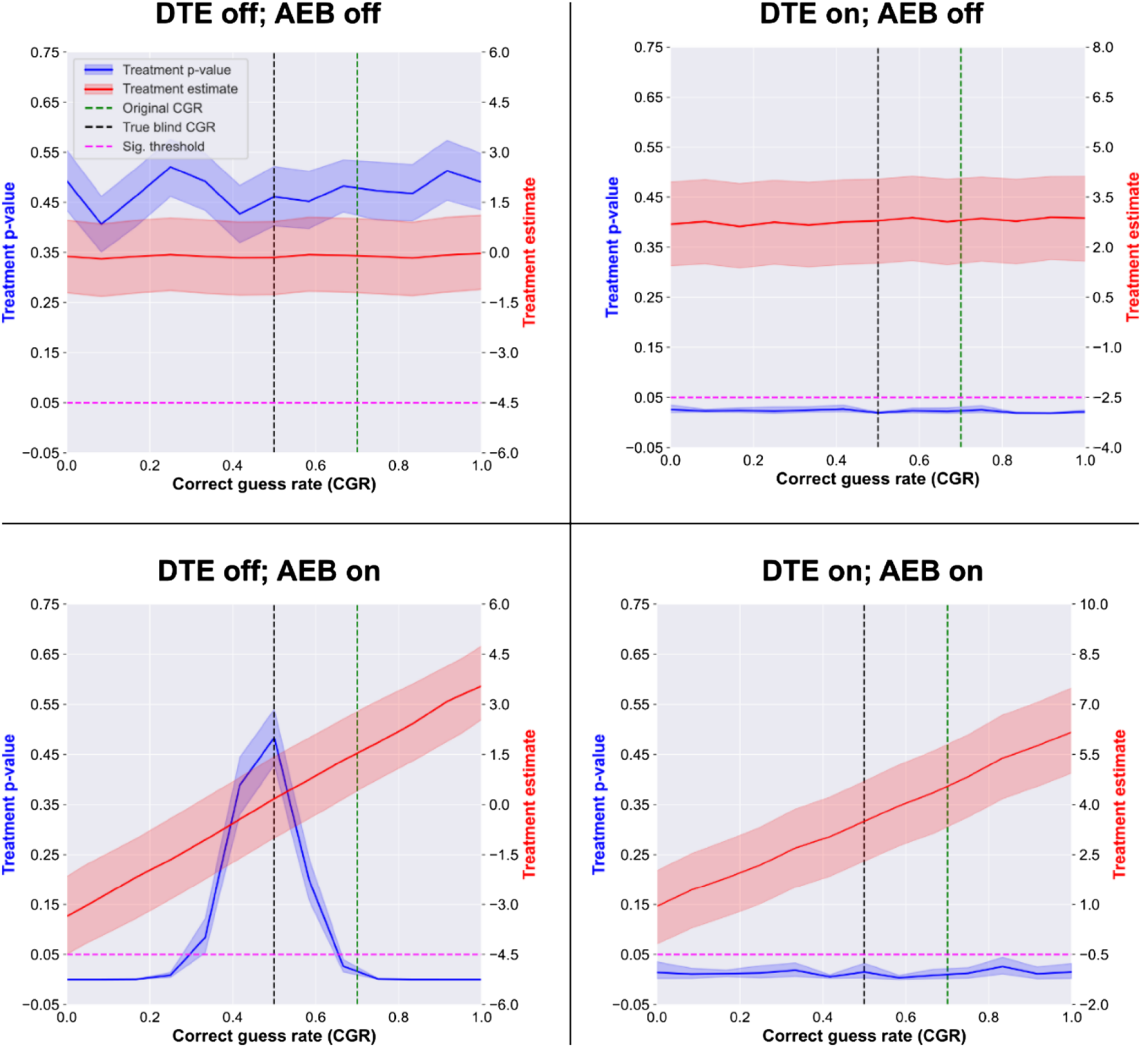
Correct guess rate (CGR) curves of the activated expectancy bias (AEB) model. Each panel shows the estimated treatment p-value (blue; scale shown on left y-axis) and effect size (red; scale shown on right y-axis), with their corresponding confidence interval, as a function of CGR. Horizontal purple dashed line represents the p = .05 significance threshold, vertical green dashed line corresponds to the simulated trial’s original CGR, while the black dashed line corresponds to a perfectly blinded trial (CGR = 0.5). The model was analyzed with 2*2 = 4 configurations of parameters, corresponding to the possibilities of the *direct treatment effect* (DTE) and *activated expectancy bias* (AEB) either being active or inactive, see Fig. 1. For the *DTE off; AEB on* case (bottom left) generates a false positive finding when CGR is not considered during analysis (green dashed line intersects p-value estimate below 0.05), but CGR adjustment recovers the lack of treatment effect (black dashed line intersects p-value estimate above 0.05). For the *DTE on; AEB on* case (bottom right), both analyses correctly identify that there is a treatment effect; however, non-CGR adjusted analysis overestimates the effect size by ~ 40%, see Table 1 for numeric results.

The model is analyzed with 2*2 = 4 parameter configurations, corresponding to the *direct treatment effec*t (DTE) and *activated expectancy bias* being active or not, see Fig. 1. Results are equivalent for the two analysis in the top two rows, however, when only the *activated expectancy bias* is active (3rd row from top), traditional analysis produces false positive findings for 78% of the simulations. Furthermore, when both *direct treatment effect* and *activated expectancy bias* are active (bottom row), traditional analysis overestimates the known true treatment effect (estimate is 5.69 points, while the true effect is 3 points), see Fig. 3 for the corresponding CGR curves.

Next, the case was analyzed where a *direct treatment effect* was active, but *activated expectancy bias* was not active (second row from top in Table 1). Non-CGR adjusted and CGR adjusted analysis identifies a significant treatment effect in 86/84% of the simulations with an average p-value of 0.032/0.036, respectively. We note that this 14%/16% false negative rate is due to the small effect used in simulations (~0.4 Hedges’ g), larger effects decrease the false negative rate of both analyses, see robustness analysis in Supplementary materials. In both analysis the treatment estimate is within 5% of the true effect.

Next, the case was analyzed where a *direct treatment effect* was inactive, but *activated expectancy bias* was active (third row from top in Table 1), i.e. a scenario where there is no true treatment effect and activated expectancy is a complete mediator of the treatment. For the traditional models, 78% of the simulated trials resulted in a false positive treatment effect. For the CGR-adjusted models, only 3% of the simulated trials produced a false positive treatment effect.

Finally, the case was analyzed where both a *direct treatment effect* and *activated expectancy bias* were active (bottom row in Table 1), i.e., a case where AEB is a partial mediator of treatment. The average *treatment* p-value was 0.001/0.041 with 99%/82% of the trials resulting a significant treatment effect for the traditional/CGR adjusted analysis, respectively. Note that the CGR adjusted analysis can only be as good to detect a treatment effect as the unadjusted analysis when only DTE is active (as the adjustment aims to remove the effect of AEB). Thus, CGR adjusted analysis detects an effect in just 4% less of the simulations (86% vs. 82%) than this best-case scenario, i.e. CGR adjustment only adds 4% to the false negative rate. Furthermore, the traditional analysis estimated the effect to be 5.69 points, while the CGR adjusted estimate was 3.04 points (the true treatment effect was 3), so traditional analysis significantly overestimated the effect due to the influence of AEB. In summary, the CGR adjusted analysis’ false negative rate is ~2-4% higher than the traditional analysis’ (rows 2&4 in Table 2), but the false positive rate is ~75% lower when AEB is present (row1&3 in Table 2). Furthermore, when a true effect is present, CGR provides a more reliable estimate of the effect size (row 4 in Table 2) as it subtracts the influence of AEB.

### Correct guess rate (CGR) adjusted analysis of the self-blinding microdose trial

Next, we advance from analyzing pseudo-experimental data to scrutinizing empirical data from the self-blinding microdose trial^24^. Using traditional, i.e. non-CGR adjusted, data analysis, statistically significant placebo-microdose differences were observed on the following scales: acute *emotional state* (PANAS; mean difference ± SE = 3.2 ± 1.3; p = 0.01**), *energy visual analogue scale VAS* (11.5 ± 2.7; p < 0.001***), mood VAS (6.4 ± 2.7; p = 0.02*), creativity VAS (6.4 ± 2.5; p = 0.01*) and post-acute depression (QIDS; − 1.2 ± 0.06; p = 0.04*).

After CGR adjustment, none of these outcomes remained significant with the exception of the energy VAS that remained significant (p ~ 0.04), but with a ~ 40% reduced effect size.

**Figure 4:**
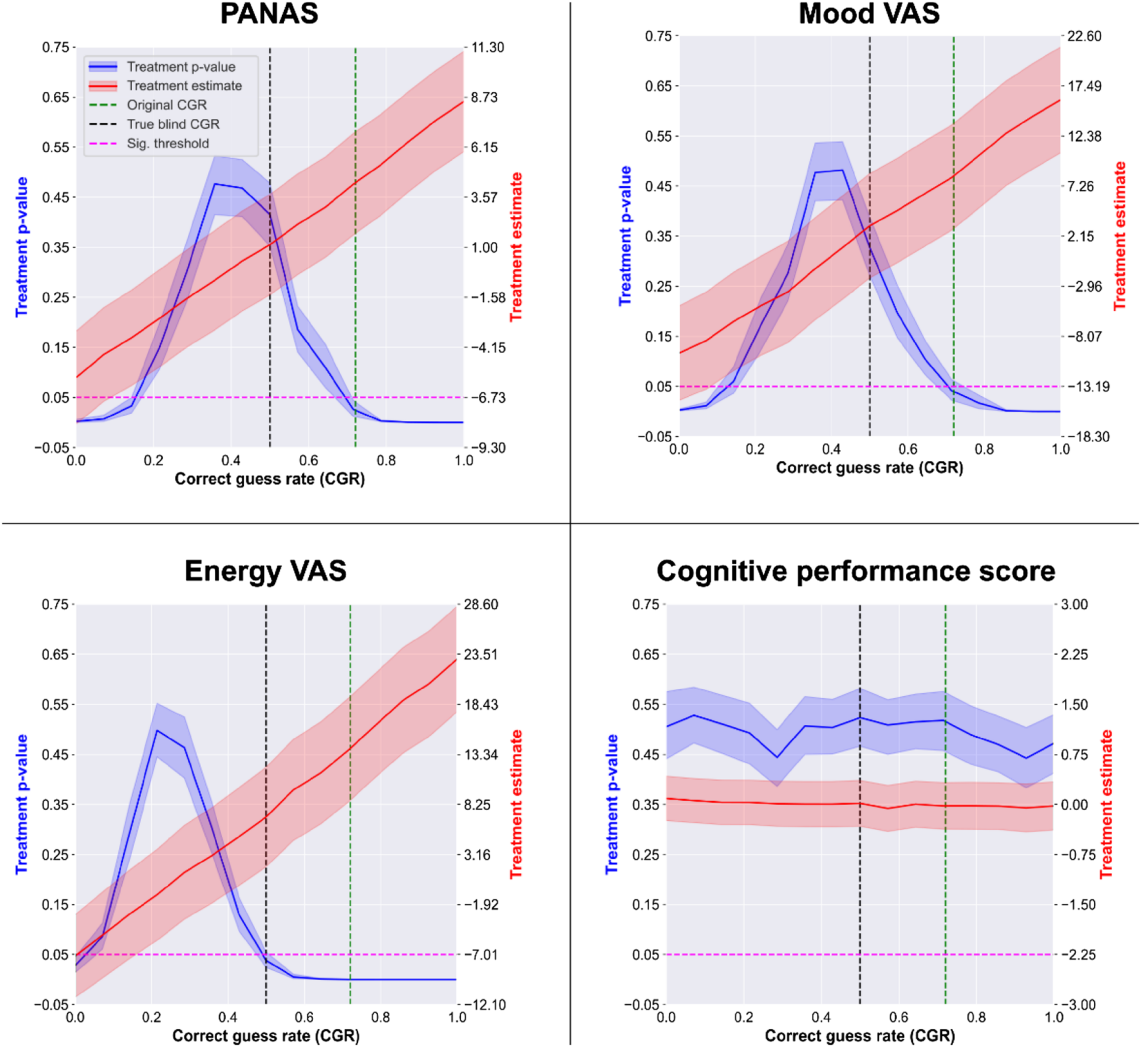
Correct guess rate (CGR) curves for self-blinding microdose trial outcomes. Each panel shows the estimated treatment p-value (blue; scale shown on left y-axis) and effect size (red; scale shown on right y-axis), with their corresponding confidence interval, as a function of CGR. Horizontal purple dashed line represents the p = .05 threshold, vertical green dashed line corresponds to the trial’s original CGR (= 0.72), while the black dashed line corresponds to a perfectly blinded trial (CGR = 0.5). Outcomes in the top row (*Positive and Negative Affection Scale* (PANAS) and *Mood visual analogue scale*) are significant according to unadjusted analysis (green dashed line intersects p-value estimate below 0.05), but become insignificant after CGR adjustment (black dashed line intersects p-value estimate above 0.05), arguing that these findings could be false positives driven by AEB. *Energy VAS* remains significant even after CGR adjustment, although the effect size is reduced by ~ 40%. This finding suggests that microdosing increases self-perceived energy beyond what is explainable by expectancy effects, although the remaining effect is small (Hedges’ g = .34). Finally, CGR adjustment has little impact on the cognitive performance score as both the p-value and the effect estimate remain close to a constant. This finding suggests that this measure is not affected by AEB, possibly because cognitive performance was not self-rated, rather measured by objective computerized tests, see Table 2 for numerical results.

This finding suggests that microdosing increases self-perceived energy beyond what is explainable by expectancy effects, although the magnitude of the remaining effect is small (Hedges’ g = 0.34). Equivalence testing for all outcomes where significance changed after CGR adjustment (i.e. *PANAS*, *QIDS*, *mood* and *creativity* VASs) with an equivalence bound equal to the average within-subject variability were significant, arguing that outcomes were equivalent in the placebo and microdose groups after the CGR adjustment, see Supplementary materials for details. See Table 2 for numeric results and Fig. 4 for the CGR curves of selected outcomes.

**Table 2:** Comparison of traditional (non-CGR adjusted) and CGR adjusted models of the self-blinding microdose trial data.

Outcome	Unadjusted models			CGR adjusted models		
	Treatment p-value	Treatment effect ± CI	Hedge’s g	Treatment p-value	Treatment effect ± CI	Hedge’s g
Emotional state (PANAS; acute)	0.01*	3.2 ± 2.6	0.32	0.43	1.1 ± 2.6	0.11
Mental well-being (WEMWBS; post-acute	0.25	1.2 ± 2.2	0.15	0.46	0.7 ± 2.2	0.08
Depression (QIDS; post-acute	0.04*	− 1.2 ± 1.1	− 0.27	0.10	− 1.1 ± 1.2	− 0.25
Anxiety (STAIT; post-acute)	0.29	− 1.6 ± 3.0	− 0.14	0.46	− 1.2 ± 3.0	− 0.1
Social connectedness (SCS; post-acute)	0.97	− 0.0 ± 1.8	0	0.48	− 0.4 ± 1.8	− 0.06
Cognitie performance (CPS; acute)	0.63	− 0.0 ± 0.2	− 0.03	0.52	0.0 ± 0.4	0.02
Energy VAS (acute)	< 0.001***	11.5 ± 5.4	0.58	0.04*	6.8 ± 5.1	0.34
Mood VAS (acute)	0.02*	6.4 ± 5.3	0.31	0.42	2.7 ± 5.4	0.13
Creativity VAS (acute)	0.01**	6.4 ± 5	0.34	0.48	2.0 ± 5.0	0.11
Focus VAS (acute)	0.60	1.4 ± 5.2	0.07	0.45	− 1.5 ± 4.9	− 0.08
Temper VAS (acute)	0.93	0.2 ± 5.8	0.01	0.42	2.1 ± 5.8	0.1

Note that for all outcomes that were statistically significant in the traditional models became insignificant after CGR adjustment with the exception of the energy VAS. These results argue that positive outcomes in the traditional analysis could be false positive findings created by AEB. Energy VAS remained significant even after CGR adjustment, although the effect size is reduced by ~ 40%. This finding suggests that microdosing increases self-perceived energy beyond what is explainable by expectancy effects, although the remaining effect is small, see Fig. 4 for CGR curves of selected outcomes.

### Treatment guess questionnaire

In the supplementary materials we included a brief, 5-items questionnaire developed to collect treatment guess and source of unblinding data. The resulting data is compatible with the current and planned future versions of the CGR curve.

## Discussion

Effective blinding distributes expectancy effects equally between treatment arms^3^. However, if blinding is ineffective, i.e. patients can deduce their treatment allocation, and if patients have a positive expectancy bias for the active arm, then expectancy effects will be no longer equally distributed and trial outcomes will be biased towards the active arm. We call this bias ‘activated expectancy bias’ (AEB), which can be viewed as a residual expectancy bias potentially present even in ‘blinded’ trials. A key consequence is that the research community needs to distinguish between *trials with a placebo-control group,* i.e., when a placebo control group is formally present in the trial*,* and *placebo-controlled trials*, where patients are genuinely blinded and thus AEB is not present. In other words, a placebo control group is necessary, but in-itself insufficient to control for expectancy effects. For example, a recent trial on LSD therapy includes *‘double-blind, placebo-controlled’* in its title, but as the manuscript describes *"only one patient in the LSD-first group mistook LSD as placebo”* (out of 18 patients), highlighting that the trial was formally blinded, but not in practice^36^. The implication is that ‘placebo-controlled’ studies are more fallible than conventionally assumed with consequences for evidence-based medicine.

Current FDA drug approval only requires two trials with statistically significant drug-placebo difference^37^, thus, the self-blinding microdose trial yielded evidence consistent with FDA approval, despite that the findings were likely false positives, driven by AEB. In our view, placebo-controlled trials should only be considered ‘gold standard’ if blinding integrity is demonstrated with empirical data. This requirement would create a new, more rigorous standard for what is ‘placebo control’. Given the high costs and low success rate of psychiatric trials^38^, there may be little appetite from industry and regulators to create such new standard, but it should be embraced by the scientific community.

We note that it is difficult to estimate how prevalent AEB is in medical trials, because blinding integrity has only been reported in 2–7% of trials^5,6,9^. To understand the prevalence of AEB, more trials need to capture blinding integrity data^39^. To aid this practice, in the supplementary materials we suggest a brief 5-items questionnaire that is compatible with the method presented here and recommend its adoption.

When the self-blinding microdose trial was analyzed traditionally, small, but significant microdose-placebo differences were observed on *emotional state, depression, mood, energy* and *creativity,* favoring microdosing^24^. After CGR adjustment, only *energy VAS* remained significant (p ~ 0.04) with a ~ 40% reduced effect size—we note that another recent trial similarly found significant increases in self-perceived energy beyond what is explainable by the placebo and expectancy effects^40^. One could argue that these negative results are false negatives; however, the consistency of the negative results across measures argues against this possibility. Furthermore, the trial had the necessary features for AEB, i.e. weak blinding and a positively biased^24^, implying that the trial is susceptible to AEB. AEB is likely to be present in other psychedelic microdose trials as well, results should be interpreted with caution, especially if evidence for effective blinding is not presented.

We hypothesize that the reported benefits psychedelic microdosing on mood and creativity can be understood as an ‘active placebo’, i.e., an intervention without medical *benefits*, but with perceivable *effects*^36,39,40^, emphasizing the difference between *effects* and *benefits*. A recent comprehensive review of microdosing concluded that: *“These findings together provide clear evidence of psychopharmacological effects. That is, microdosing is doing something. A key question for researchers is whether the effects of microdosing have clinical or optimization benefits beyond what might be explained by placebo or expectation.”*^41^. In short, microdosing leads to *perceivable effects*, for example by the heightened energy levels, explaining why CGR is universally high across trials^21,24,40^, but at this point none of these effects seem to be related improved mental health. If our hypothesis is correct, then, either improved blinding or a sample without positive expectancy would nullify the observed benefits of microdosing by nullifying AEB. An alternative possibility is that microdosing is only effective at doses where blinding integrity cannot be maintained due to conspicuous subjective effects, such as in the case of psychedelic macrodosing^42^. In this scenario the possibility of effective placebo control is abandoned and efficacy beyond expectancy needs to be established outside of blinded trials. Arguments for the merit of alternative trial designs to assess the efficacy of psychedelics have been made before^43^, for example mechanistic studies could also help to establish the causal effect of treatment. Recently, arguments against the value of placebo control have been raised in psychedelic trials^44^. This article remains neutral on this issue, it merely insists that if a trial is called ‘placebo controlled', then it should really control for the placebo effect and not just have a ‘placebo group'.

Our arguments above assume that the high CGR is explained by *malicious unblinding*, i.e. positive treatment expectancy drives the positive outcomes, rather than *benign unblinding*, i.e. patients correctly guess their treatment due to noticeable health improvements^45^. If unblinding is benign, then CGR adjustment could lead to false negative findings due to collider bias^46^ (currently Fig. 1 represents *malicious unblinding*, for *benign unblinding* PT → TE → OUT would need to be replaced with OUT → PT). Accordingly, investigators need to carefully assess the source of unblinding prior to using our method. To facilitate this assessment, our questionnaire in the supplementary materials captures this source of unblinding information.

What was the source of unblinding in the self-blinding psychedelic microdose trial? Two lines of evidence point towards that it was the perceptual/side effects rather than efficacy, corresponding to malicious unblinding. First, 55% reported that the primary cue to formulate their treatment guess was ‘*body/perceptual sensations’*, such as muscle tension (58%) and stomach discomfort (27%), in contrast only 23% reported ‘*mental/psychological benefits’*. Secondly, among participants who were assessed under both placebo and microdose conditions, the mean placebo-microdose difference on the positive / negative affect subdomains of the PANAS was 2.1/0.8. In a recent study without any intervention, the mean temporal intra-individual difference, i.e. the within-subject day-to-day variability, of the same subdomains was ~ 10/~ 6^47^. Thus, the natural within-subject variability is ~ 500–750% larger than the mean placebo-microdose difference, arguing that the effect is too small to be noticeable.

### Limitations

CGR adjustment relies on binary treatment guess data from patients, however, treatment belief is a complex construct that cannot be reduced to a single binary variable. We focused on binary guess data due to its availability and note that even this imperfect data is rare to find. Treatment guess could be better characterized if guess confidence was also rated. Such confidence data would allow to distinguish between those who truly identified their drug condition (high confidence guess) versus those who guess correctly by chance (low confidence guess).

In our analysis, we treat the source of unblinding as a binary variable, either being only benign or malicious. A more realistic scenario is that for some patients, both efficacy and non-specific effects contribute to their guesses. Relatedly, our assessment on the source of unblinding is based on retrospective self-reports, that cannot provide conclusive evidence on causation.

Our AEB model assumes linear addition of the direct treatment and the activated expectancy effects to estimate the total effect, however, these effects may not be additive for all circumstances^48^.

The CGR curve relies on resampling the observed data, thus, the resulting data cannot be considered experimentally randomized, and as a consequence confounding variables may not be equally distributed. Despite the KDE approximation of each strata, practically some datapoints may appear multiple times in the pseudo-experimental samples, potentially increasing the error rate due to dependent observations. The error rate of our methodology is a function of the sample characteristics, generally, the smaller the sample, the more extreme the CGR and the smaller the effects are, the less reliable the results will be. In a range of these parameters that mimics microdosing and antidepressant trials (n ~ 200, CGR ~ 0.7, treatment effect ~ 0.4 Hedges’ g), our method has comparable false negative rate as traditional, non-CGR adjusted analysis. However, when AEB is present CGR adjusted analysis has a much lower false positive rate and a more reliable estimate of the true effect size compared to non-CGR adjusted analysis. The error rate of our methodology can be higher in other contexts, in particular if the sample is small. Researchers wishing to use CGR adjustment should first run simulations to determine whether CGR produces acceptable error rates for the parameters of their data and the application in mind. For the limitations listed above, our CGR adjustment is inferior to results from a truly blind RCT, its value lies that it can provide an approximate answer when achieving ideal blinding is difficult or impossible.

CGR adjustment can be viewed as an example of a resampling method to overcome the challenges of imbalanced data. Here we present only a particular solution to this problem and not a systematic exploration of how rebalancing of the data can be achieved.

Finally, our data on microdosing was obtained from a self-selected healthy sample. Microdosing may be effective for certain conditions in a clinical population, in domains we did not assess, if used at higher doses or longer time periods or when it is co-administered with a behavioral therapy, such as cognitive training.

## Supplementary Information

Supplementary Information.

## Data Availability

The data and software used here is available for scientific and research purposes at https://github.com/szb37/CorrectGuessRateCurve. The repository contains a conda computational environment, the data analyzed and scripts to reproduce all figures and major statistical findings described here.
