# Supplementary material for: The difference between ‘placebo group’ and ‘placebo control’: a case study in psychedelic microdosing
Source: Sci Rep. 2023 Jul 26;13:12107. doi: 10.1038/s41598-023-34938-7 (PMC10371989; doi:10.1038/s41598-023-34938-7)
Supplement: Supplementary file 1 — Supplementary Information. [file 41598_2023_34938_MOESM1_ESM.pdf]

Supplementary materials

Guess of Treatment questionnaire

The questionnaire below was developed to collect treatment guess and source of unblinding data. The resulting data is compatible with the current and planned future versions of the CGR curve. The wording is optimized for psychedelic interventions, but can be adopted to other treatments, see notes below. This questionnaire has not been validated; we seek opportunities for collaboration to validate this questionnaire.

When to administer the questionnaire? Generally, we recommend to administer it as the last questionnaire at the primary endpoint(s). Thus, if the trial has one/few drug session(s) and the primary endpoint is after the drug administration(s), as typical in trials of psychedelic assisted psychotherapy, then we recommend to administering this questionnaire once at the primary endpoint. If the trial has many dosing sessions and the primary outcomes are measured after each drug administrations, then we recommend administering this questionnaire after each primary measure.

Notes:

- Response is mandatory for all items, except question 5.
- For Question 1, add/change response options as appropriate for your study
- For Question 1, if there are multiple dosing sessions, consider adding to the *end 'about the treatment you received the last dosing session'*
- Questions 2, 3 and 4 are measured on a continuous visual analogue scale (VAS) with anchor points at 0%/50%/100% as shown below.
- Questions 3 & 4 are dependent on the response to Question 1. If the response to Question 1 was an active treatment, then use questions 3a and 4a, otherwise use questions 3b and 4b.
- If you have a non-psychedelic intervention, then the wording in () in Questions 3&4, both *a* and *b* versions, should be adopted such that the side effects description matches the intervention in your trial
- If in question 2 the responder indicats that the guess was random (0% confidence), then skip questions 3-5

Questions:

1. Please indicate your best guess about the treatment you received:
   - Psilocybin
   - Placebo

2. Please rate your confident in your guess:

No confidence, guess is random | Moderately confident | Completly confident

3a. Please rate the following statement: my guess is based on the side effects and/or perceptual drug effects (e.g. muscle tension, visual distortions etc.) that I attribute to receiving an active drug.

Not true at all | Moderately true | Completly true

4a. Please rate the following statement: my guess is based on health improvements that I attribute to receiving an active drug.

Not true at all | Moderately true | Completly true

3b. Please rate the following statement: my guess is based on the lack of side effects and/or perceptual drug effects (e.g. muscle tension, visual distortions etc.) that I attribute to receiving placebo.

Not true at all | Moderately true | Completly true

4b. Please rate the following statement: my guess is based on the lack of health improvements that I attribute to receiving placebo.

Not true at all | Moderately true | Completly true

5. If factors other than side effects and/or health improvements helped you to formulate your guess, please explain below.

- [Optional text box response]

Strata sample size calculation for CGR adjusted sample

This example illustrates how starata sample sizes are calculated. Let's assume an experiment has the following sample sizes for each strata:

$n_{PL/PL}$=65,

$n_{AC/PL}$=35,

$n_{PL/AC}$=25,

$n_{AC/AC}$=75,

resulting in CGR=0.7 with $n_{AC/AC}/n_{PL/PL}$~1.15 and $n_{AC/PL}/n_{PL/AC}$~1.4.

Then the CGR adjusted pseudo-experimental sample would have the following sample sizes:

$n_{PL/PL}$ = (200/2)*(65/(65+75)) ~ 46.4 ~ 46,

$n_{AC/PL}$ = (200/2)*(35/(35+25)) ~ 58.3 ~ 58,

$n_{PL/AC}$ = (200/2)*(25/(35+25)) ~ 41.6 ~ 42,

$n_{AC/AC}$ = (200/2)*(75/(65+75)) ~ 53.6 ~ 54,

resulting in CGR=0.5 with $n_{AC/AC}/n_{PL/PL}$~1.17 and $n_{AC/PL}/n_{PL/AC}$~1.38, i.e. the ratios $n_{AC/AC}/n_{PL/PL}$ and $n_{AC/PL}/n_{PL/AC}$ are approximately the same as in the original data

Treatment guess data in the self-blinding microdose trial

Participants were asked to guess which type of capsule they had taken that day during the dose period (for days when capsule was taken). This guess was a forced binary choice between microdose and placebo options. At the end of the post-acute test sessions, participants were asked separately to guess whether the current week was a microdose or a placebo week. For the acute outcomes the 'daily guess' was used, for the post-acute outcomes the 'weekly guess' was used.

We note that participants prepared 8 microdose and 24 placebo doses, so the MD:PL doses ratio was 1:3. A random guesser with this knowledge is expected to have a CGR=0.67, in contrast, if this knowledge is not considered, then CGR=0.5 is expected. To resolve this question, we retrospectively surveyed participants, asking them *'Did you consider the ratio of prepared placebo/microdose capsules when guessing your capsule type?'* with response options of '*Yes*' and '*No*'. All 61 responses received were *'No'*, hence the random guesser's CGR is treated as 0.5.

Equivalence testing

We performed equivalence testing for the outcomes where the non-CGR adjusted analysis revealed significant between-condition differences that became insignificant in the CGR-adjusted analysis (*PANAS, QIDS, mood* and *creativity* VASs) to demonstrate that these results are likely to be ‘true false positives’. We used the *two one sided test* (TOST) procedure (Lakens et al., 2020) as implemented by the TOSTER package in R. For the equivalence bound, we choose the average within-subject standard deviation for participants in the placebo group, i.e. the average variability from measurement to measurement in the absence of an active drug. Effects smaller than this are arguably too small to make a meaningful difference.

We run the TOST test on all 100 CGR adjusted samples. For the *PANAS*, *mood* and *creativity* outcomes, on all samples the equivalence test was significant, while for QIDS, the equivalence test was significant for 84% of the samples, arguing that effects larger than the equivalence bound can be rejected after CGR adjustment.

Model parameters

Parameter	(μ_{NH}, σ_{NH})	$(\mu_{DTE}, \sigma_{DTE})$	$(\mu_{AEB}, \sigma_{AEB})$	p_{TRT}	p_{CG}
DTE off, AEB off	(10,4)	(0,0)	(0,0)	0.5	0.7
DTE on, AEB off	(10,4)	(3,6.2)	(0,0)	0.5	0.7
DTE off, AEB on	(10,4)	(0,0)	(7.7,6.2)	0.5	0.7
DTE on, AEB on	(10,4)	(3,6.2)	(7.7,6.2)	0.5	0.7

Supplementary table 1: AEB model parameters, see Figure 1 for the network structure and equations defining the models. Effect parameters are higher for the AEB pathway as this pathway only effects a subset of the data, specifically the correct guess in the active arm strata.

Robustness analysis

To show that the results presented in Table 1, where the CGRC is applied to data generated by the AEB model, do not require fine tuning of parameters, we rerun each model configuration with either increasing / decreasing the treatment effect, total sample or the correct guess rate. These results are qualitatively consistent with Table 1, demonstrating the robustness of the method.

Model configuration	Model parameters				Non-CGR adjusted models	CGR adjusted models
	Correct guess rate (CGR)	Sample size	Direct *trt.* effect (points)	Direct *trt.* effect (Hedges' g)	Proportion with sig. *treatment* p-value	Proportion with sig. *treatment* p-value
DTE off, AEB off	0.7	260	0	0	0.05	0.06
DTE off, AEB off	0.8	230	0	0	0.04	0.05
DTE off, AEB off	0.6	230	0	0	0.05	0.05
DTE off, AEB off	0.7	200	0	0	0.05	0.06
DTE on, AEB off	0.7	260	3	0.4	0.88	0.87
DTE on, AEB off	0.7	230	3	0.45	0.9	0.87
DTE on, AEB off	0.8	230	3	0.4	0.86	0.81
DTE on, AEB off	0.6	230	3	0.4	0.86	0.85
DTE on, AEB off	0.7	230	3	0.35	0.83	0.8
DTE on, AEB off	0.7	200	3	0.4	0.82	0.8
DTE off, AEB on	0.7	260	0	0	0.81	0.03
DTE off, AEB on	0.8	230	0	0	0.78	0.04
DTE off, AEB on	0.6	230	0	0	0.79	0.02
DTE off, AEB on	0.7	200	0	0	0.76	0.05
DTE on, AEB on	0.7	260	3	0.4	0.99	0.85
DTE on, AEB on	0.7	230	3	0.45	0.99	0.86
DTE on, AEB on	0.8	230	3	0.4	0.99	0.83
DTE on, AEB on	0.6	230	3	0.4	0.99	0.84
DTE on, AEB on	0.7	230	3	0.35	0.97	0.81
DTE on, AEB on	0.7	200	3	0.4	0.97	0.81

Supplementary table 2: robustness analysis.

Supplementary references

Lakens, D., McLatchie, N., Isager, P. M., Scheel, A. M., & Dienes, Z. (2020). Improving Inferences About Null Effects With Bayes Factors and Equivalence Tests. *The Journals of Gerontology. Series B, Psychological Sciences and Social Sciences*, *75*(1), 45–57. https://doi.org/10.1093/geronb/gby065
